# Supplementary material for: Phonon downconversion to suppress correlated errors in superconducting qubits
Source: Nat Commun. 2022 Oct 28;13:6425. doi: 10.1038/s41467-022-33997-0 (PMC9616905; doi:10.1038/s41467-022-33997-0)
Supplement: Supplementary file 1 — Supplementary Information [file 41467_2022_33997_MOESM1_ESM.pdf]

Supplementary Information: Phonon downconversion to suppress correlated errors in superconducting qubits

V. Iaia,[1,*] J. Ku,[1,*] A. Ballard,[1] C. P. Larson,[1] E. Yelton,[1]
C. H. Liu,[2] S. Patel,[2] R. McDermott,[2] and B. L. T. Plourde[1,†]

[1]*Department of Physics, Syracuse University, Syracuse, New York 13244-1130*
[2]*Department of Physics, University of Wisconsin-Madison, Madison, Wisconsin 53706*

(Dated: September 29, 2022)

SUPPLEMENTARY NOTE 1: DEVICE FABRICATION

Both the non-Cu and Cu chips are fabricated on high-resistivity ($> 10\,\mathrm{k}\Omega$-cm) 100-mm Si wafers; for the Cu chip, the wafer is double-side polished to facilitate the deposition and patterning of the Cu reservoirs. Initially the wafer is put through a standard RCA clean process and then submerged in a buffered-2% per vol. HF bath to remove native oxides immediately before sputter-deposition for the non-Cu (Cu) chip of a 55-nm (80-nm) thick Nb film on the top surface of each wafer. We pattern the Nb films using a deep-UV photostepper to define the ground plane, feedline, readout resonators, qubit islands, charge-bias lines, and injector junction pads followed by a dry etch using BCl_3, Cl_2, and Ar in an ICP etcher. In the case of the non-Cu devices, we proceed with the wafer to the Josephson junction definition step, while for the Cu devices, we next fabricate the Cu reservoirs.

After stripping the base layer resist with a TMAH hot strip bath, we then coat the surface with the Nb pattern using a thick photoresist layer (SPR-220-3.0) to protect the Nb during the subsequent backside processing for the Cu reservoirs. We then deposit a metal seed layer on the back side of the wafer using electron-beam evaporation of Ti (20 nm) at a deposition rate of 1Å/s followed by Cu (100 nm) deposited at 2Å/s.

For the Cu reservoirs, we deposit Cu on the wafer back side with an electrodeposition process by submerging our wafer into a copper sulfate and sulfuric acid solution. We grow a 10-μm thick Cu film on top of the seed layers at a rate of $\sim$3.3 μm/hr using an alternating current deposition mode. A test film grown with the same parameters and patterned into a narrow strip using Kapton tape was measured to have RRR $\sim$42. The islands were defined with a lattice of partial 50-μm-wide dicing saw cuts through the Cu film into the back side of the wafer, with the cuts extending $20\,\mu$m into the back surface of the Si, resulting in island areas of $(200\,\mu\mathrm{m})^2$. After the Cu islands are fabricated, all resist is stripped in a TMAH hot strip bath.

For both wafers, the Josephson junctions are then defined with a conventional double-angle shadow-evaporation process using 100 keV electron-beam lithography of a PMMA/MMA bilayer resist stack. After an *in situ* ion mill cleaning step to remove native oxide from the Nb surface at the contact points to the junction electrodes, the junctions are formed with electron-beam evaporation of Al. The bottom (top) junction electrode is 40 (80) nm thick.

SUPPLEMENTARY NOTE 2: DEVICE LAYOUT

Following the fabrication, the wafers are diced into chips that are $(8\ \mathrm{mm})^2$. The coplanar waveguide feedline runs across the middle of the wafer, with the 1/4-wave readout resonators for each qubit inductively coupled to the feedline. A full-chip layout can be seen in Supplementary Fig. 1, along with close-up views of each qubit, the non-Cu injector junction, and the Cu island pattern on the back side of the Cu chip. Editing of the individual micrographs to obtain the full-chip image is described in Supplementary Note 14. The locations of the qubits measured in the experiment relative to the injector junctions used for controlled QP poisoning, as well as the inter-qubit separations, are indicated in Supplementary Fig. 1(g-i).

SUPPLEMENTARY NOTE 3: DEVICE AND MEASUREMENT SETUP

Measurements on both the non-Cu and Cu chips are performed on the same dilution refrigerator cooldown running at a temperature below 15 mK. The Al sample boxes for both chips are mounted on the same cold-finger inside a single Cryoperm magnetic shield. A Radiall relay switch on the output lines of the two devices allows us to switch between measurements of one chip or the other. Supplementary Fig. 2 details the configuration of cabling, attenuation, filtering, and shielding inside the cryostat, as well as the room-temperature electronics hardware for

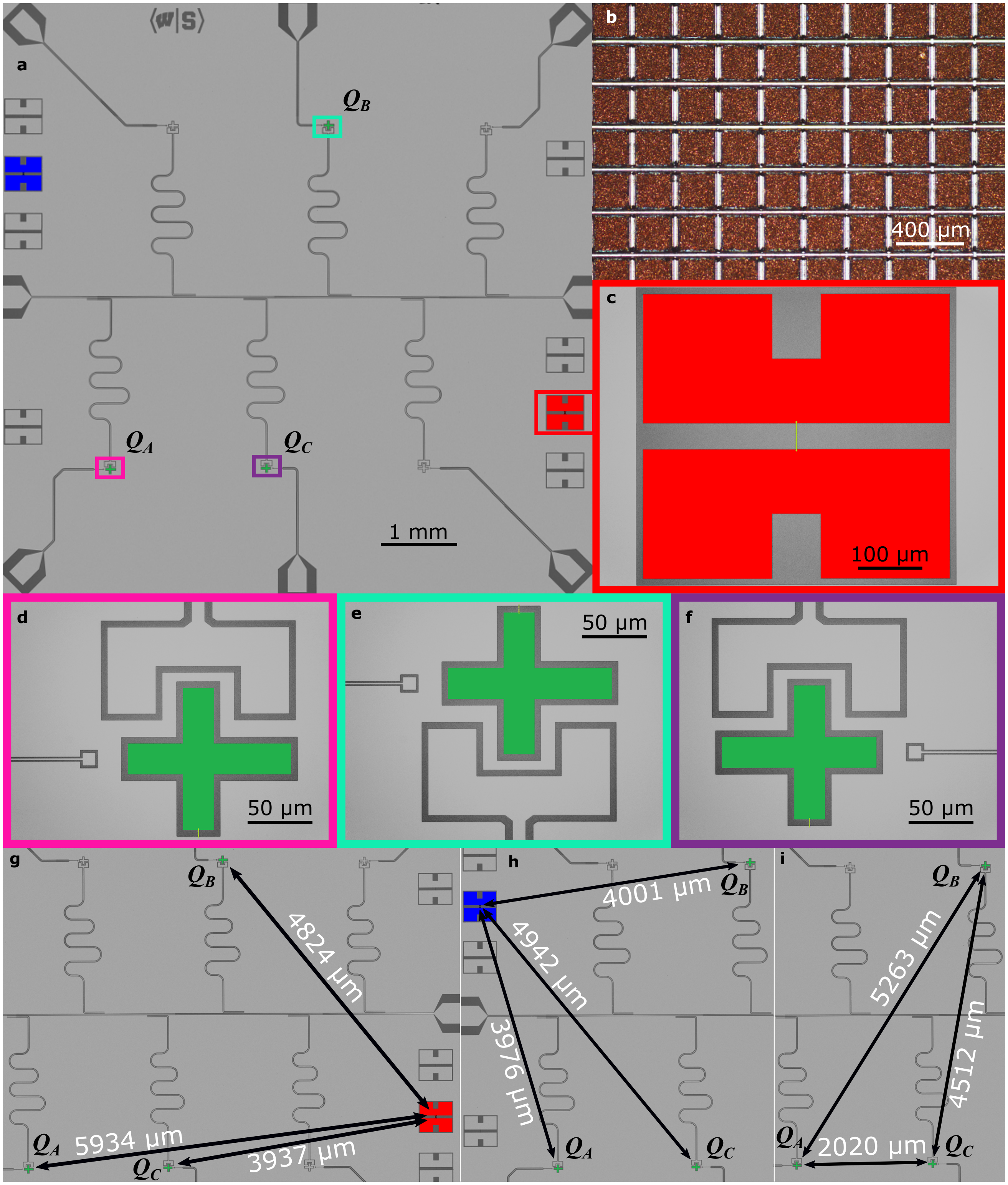

Supplementary Figure 1. **Optical micrographs of devices.** (a) Stitched composite image of the device layer of the chip (see Supplementary Note 14). (b) Cu islands on back side of the Cu chip. (c) Close-up view of the injector junction used for the non-Cu chip. Nb pads are colored in red and Al junction is highlighted yellow. (d,e,f) Close-up images of qubits ($Q_{A,B,C}$). Nb island is colored green, and Al junction electrodes are highlighted in yellow. Qubit distances from injector junction on the (g) non-Cu chip, (h) Cu chip, and (i) interqubit spacing for both chips.

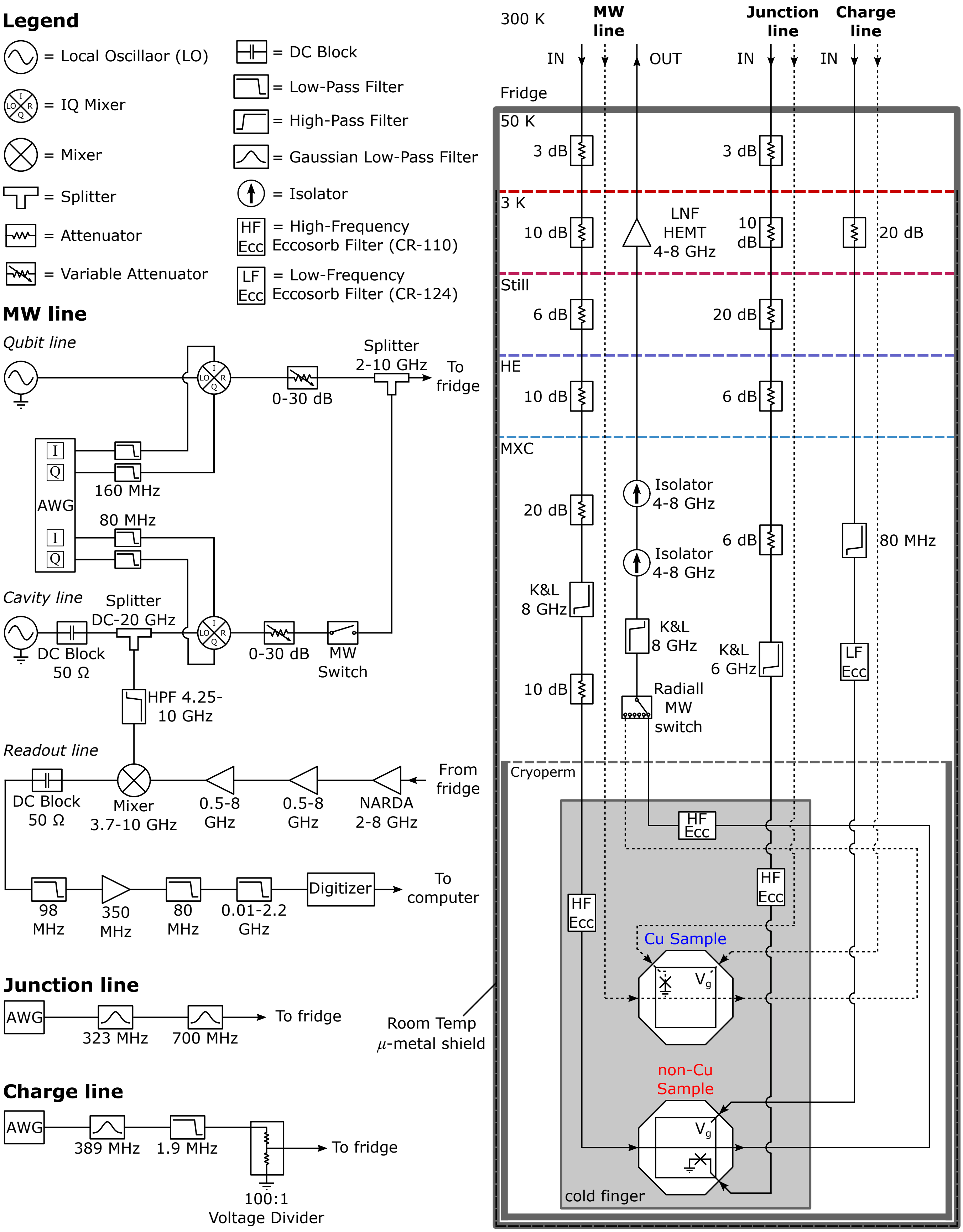

Supplementary Figure 2. **Experimental configuration.** Wiring diagram for room-temperature components on the left and cryogenic wiring on the right with different temperature stages indicated. Cryoperm magnetic shield and mixing chamber (MXC) shields have an IR-absorbent coating. All filters are LPF unless stated otherwise. The dotted lines in the fridge represent the MW, junction, and charge lines for the Cu chip and are configured identically to the corresponding lines for the non-Cu chip.

control and readout. The inner surfaces of the Cryoperm magnetic shield and the mixing chamber shield were both coated with an infrared-absorbent layer [1]. For the charge biasing of the qubits, wiring limitations on our dilution refrigerator prevented us from connecting to all of the bias traces on the chips. For the non-Cu chip, charge bias lines are connected to Q_B and Q_C; for the Cu chip, there is only a bias connection to Q_A.

SUPPLEMENTARY NOTE 4: DEVICE PARAMETERS

Supplementary Table 1 lists relevant qubit parameters for both chips, including the qubit transition frequency f_{01}, the readout resonator frequency f_{RO}, the peak-to-peak maximum charge dispersion $2\delta f$, the mean and standard deviation from repeated baseline T_1 measurements, and the E_J/E_c ratios. During the junction fabrication, the same double-angle evaporation process is used for the injector and qubit junctions, and thus all junctions on a chip have nominally the same critical current density. For each device, one of the junctions around the perimeter of the chip is connected to a 50-Ω bias lead to use as the injector junction (indicated by color highlighting in Supplementary Fig. 1); the injector junction for each chip is ∼3 times the area of the qubit junctions. Because the junctions on the Cu and non-Cu chips were processed separately, the critical current densities on the two chips are slightly different. For the injector junctions, $R_n = 3.5$ (3.0) kΩ for the non-Cu (Cu) chips. The qubit junctions were all designed to have the same area and, based on witness junctions on the same chip written with the same area, had normal resistance of $R_n = 12.2$ (10.8) kΩ for the non-Cu (Cu) chips.

Qubit Parameters							
Device	Qubit	f_{01}(GHz)	f_{RO}(GHz)	T_1(μs)	$\sigma(T_1)$(μs)	$2\delta f$(MHz)	E_J/E_c
non-Cu	Q_A	4.6555	6.0431	34	10	3.743	24
	Q_B	4.7363	6.1506	20	2	3.201	26
	Q_C	4.8408	6.229	16	2	4.631	25
Cu	Q_A	4.9959	6.3977	16	3	1.878	29
	Q_B	5.2536	6.4868	21	5	1.146	32
	Q_C	5.3190	6.5963	13	4	1.938	31

Supplementary Table 1. **Qubit parameters for both non-Cu and Cu samples.**

SUPPLEMENTARY NOTE 5: DETAILS OF $\Delta\Gamma_1$ MEASUREMENTS

For measurements of enhancements to the qubit relaxation rate following pulsing of the injector junction, in the main paper we present measurements of $\Delta\Gamma_1$ for Q_C on the non-Cu chip and Q_B for the Cu chip. In this section, we compile these measurements for the other qubits, and show that the response of the other qubits on each chip is consistent with the representative measurements in the main paper.

Supplementary Fig. 3(a) contains measurements of $\Delta\Gamma_1$ vs. the delay between the 10-μs injection pulse and the X pulse for the relaxation measurement for all three qubits on both the non-Cu and Cu chips. In Supplementary Fig. 3(b), we plot the same data for the three qubits on the non-Cu chip on a semilog scale. The black dashed line corresponds to a characteristic timescale of 60 μs for injected phonons to leave the chip following the phonon arrival peak. Error bars on $\Delta\Gamma_1$ values, here and in Fig. 2 in the main paper, are calculated from fit errors with 95% confidence intervals from T_1 fits with contributions added in quadrature.

Supplementary Fig. 4 contains plots of $\Delta\Gamma_1$ vs. V_b for a delay of 30 μs for all three qubits on both chips. In Supplementary Fig. 5, we plot the same type of measurements but with a delay of 100 ns. In this second case, the antenna-resonance peaks from the photonic coupling to the Josephson radiation emitted by the injector junction are enhanced, while the remaining phononic poisoning is somewhat lower, as not all of the injected phonons have reached the qubit yet. The change in reduced QP density in the qubit junction leads, $\Delta x_{\rm qp}$, that is plotted on the right axes can be calculated from $\Delta\Gamma_1$ as $\Delta x_{\rm qp} = \pi\Delta\Gamma_1/\sqrt{2\Delta_{\rm Al}\omega_{01}/\hbar}$ [2], where ω_{01} is the qubit transition frequency.

When the injection pulse amplitude is below $2\Delta_{\rm Al}$, we observe only minimal reduction in T_1; there is still some non-zero, but small, poisoning in this regime because our junction biasing scheme still permits the injector junction to undergo relaxation oscillations for small bias voltages [3], where the junction can momentarily switch out to the gap before retrapping.

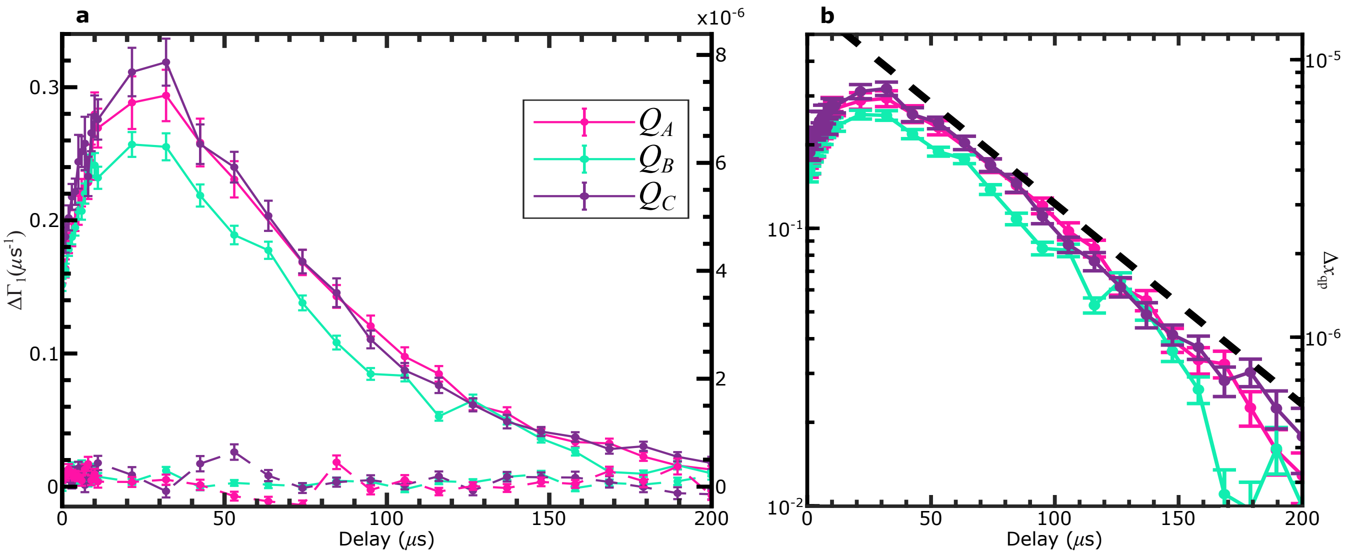

Supplementary Figure 3. **Enhancement of relaxation rate with controlled poisoning.** (a) Plot of $\Delta\Gamma_1$ vs. delay between injection pulse and X pulse for T_1 measurement for all three qubits on both chips at V_b = 1 mV where the Cu qubits are indicated by dashed lines joining the data points, while solid lines indicate the non-Cu qubits; the color labeling for each qubit is shown in the legend. (b) Plot of the same non-Cu data as in (a) plotted on a semilog scale; black dashed line indicates a characteristic decay time constant of 60 μs. The error bars on $\Delta\Gamma_1$ values represent 95% confidence intervals from T_1 fits.

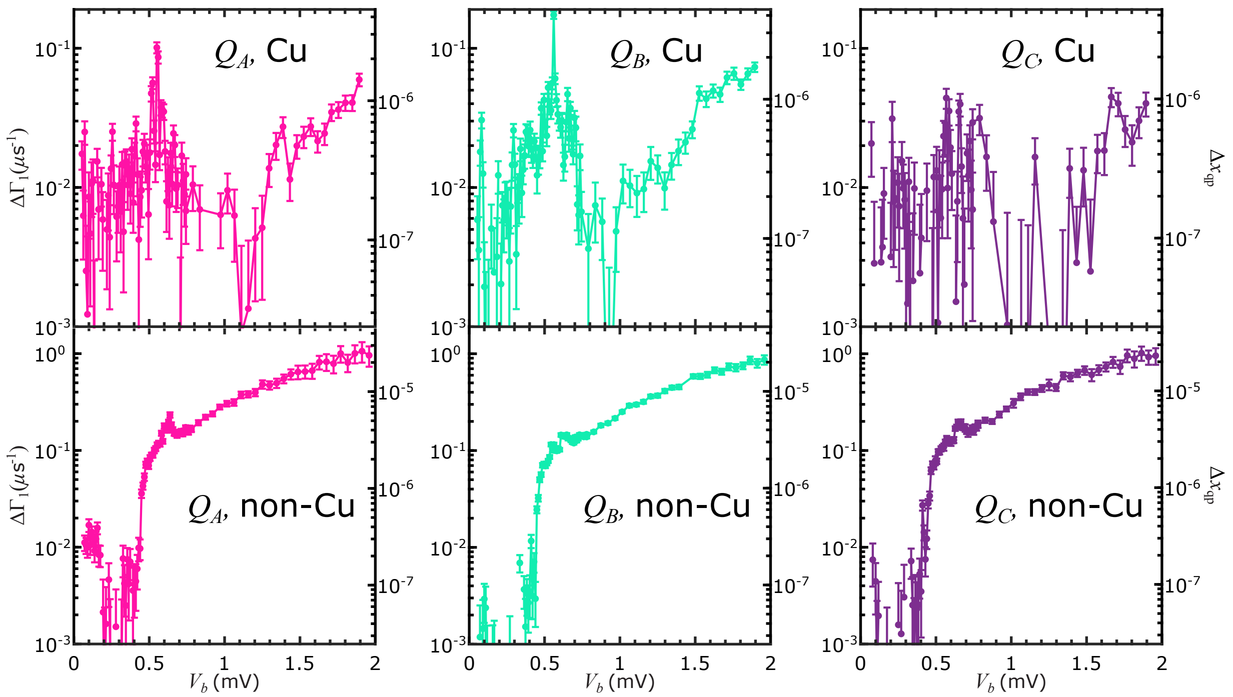

Supplementary Figure 4. **Enhancement of relaxation rate with controlled poisoning vs. injection amplitude.** Plots of $\Delta\Gamma_1$ vs. V_b for a 30-μs delay for all qubits on the Cu sample (top row) and for the non-Cu sample (bottom row). The error bars on $\Delta\Gamma_1$ values represent 95% confidence intervals from T_1 fits.

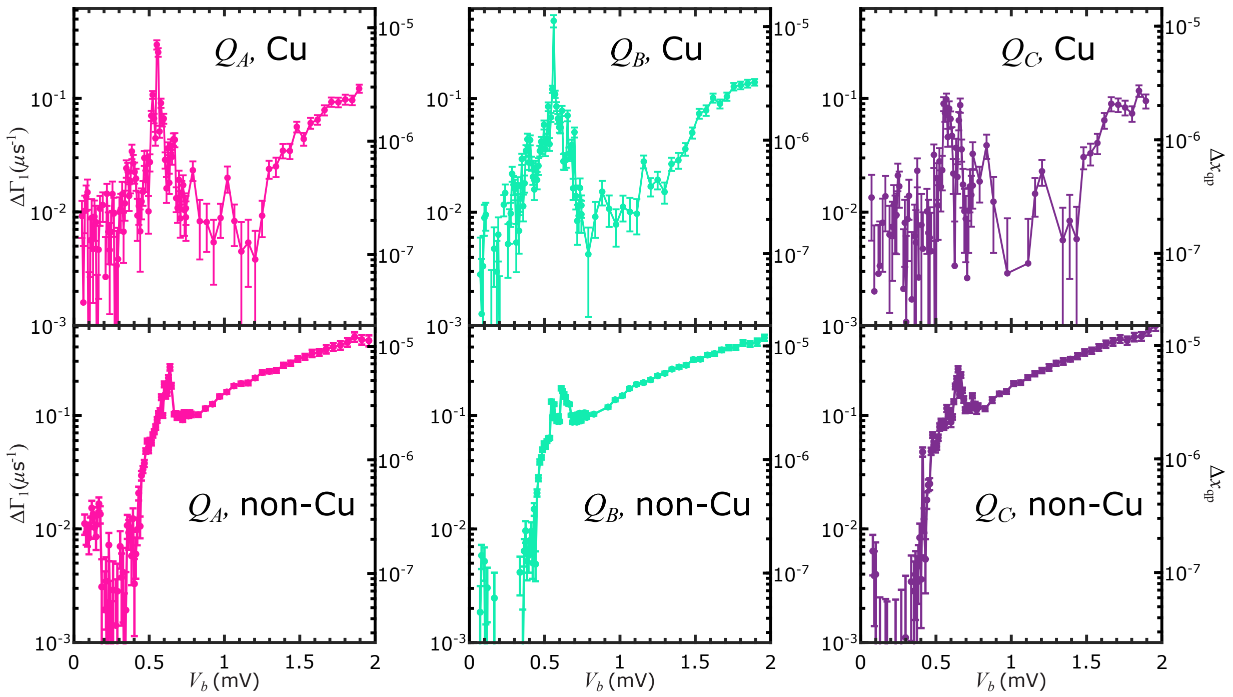

Supplementary Figure 5. **Enhancement of relaxation rate with controlled poisoning vs. injection amplitude.** Plots of $\Delta\Gamma_1$ vs. V_b with a 100-ns delay for all qubits on the Cu sample (top row) and for the non-Cu sample (bottom row). The error bars on $\Delta\Gamma_1$ values represent 95% confidence intervals from T_1 fits.

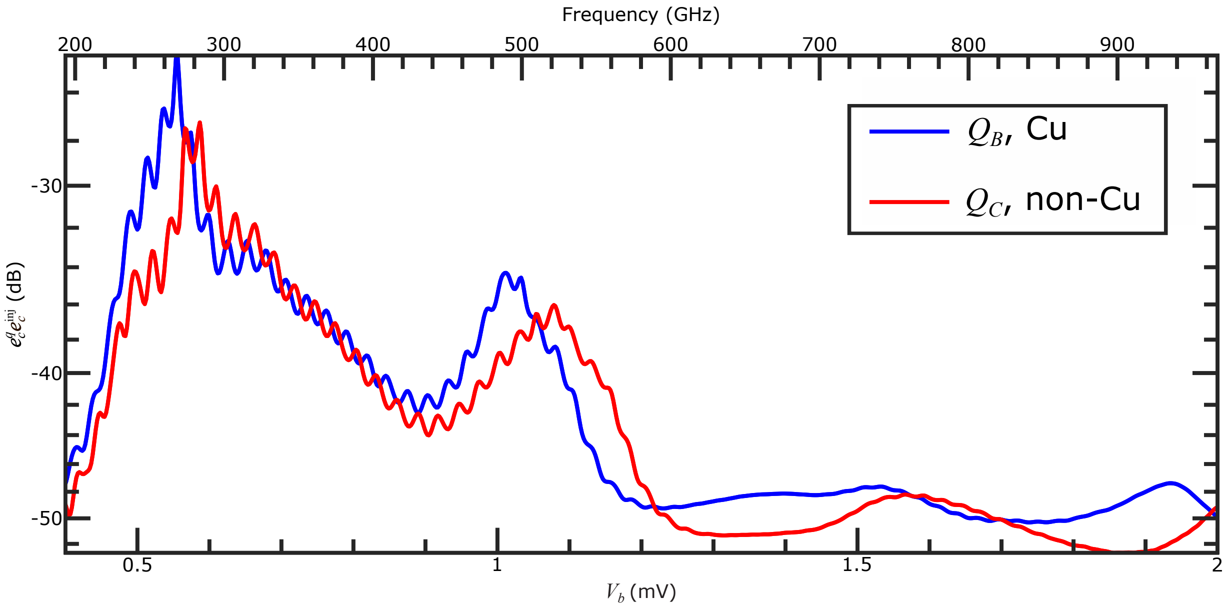

Supplementary Figure 6. **Antenna mode simulations.** Plot of $e_c^q e_c^{\mathrm{inj}}$ vs. V_b for Q_C on non-Cu chip and Q_B on Cu chip, as described in the text; scale on upper axis converted to Josephson frequency of emitted radiation.

SUPPLEMENTARY NOTE 6: ANTENNA-MODE SIMULATIONS

As described in the main paper, voltage bias of the injector junction will also induce poisoning from the emission of Josephson radiation. For a pulse amplitude V_b, the Josephson radiation will have frequency V_b/Φ_0, where $\Phi_0 \equiv h/2e$ is the magnetic flux quantum; h is Planck's constant and e is the electron charge. Such electromagnetic radiation can be resonantly absorbed by qubit structures acting as antennas, with typical resonant frequencies in the hundreds of GHz range. The absorbed radiation can then drive high-frequency currents through the qubit junction and generate QPs, as described recently in Ref. [4, 5]. A related photon-based QP poisoning mechanism was considered in Ref. [6].

In order to model the spurious qubit antenna resonances on our devices, we follow the analysis in Ref. [4, 5] and compute the radiation impedances of the injector junction and the qubit structure with a finite-element simulation using CST Microwave [7].

With the critical current values for the injector and qubit junctions extracted from the on-chip witness junction measurements, we calculate the product of the coupling efficiencies to free space for the injector junction e_c^{inj} and the qubit junction e_c^q. In Supplementary Fig. 6, we plot this product as a function of the injector junction pulse amplitude V_b for both Q_B on the Cu chip and Q_C on the non-Cu chip. The fundamental peaks in the simulation for both qubits match the measured antenna resonances from $\Delta\Gamma_1$ in Fig. 2(b) in the main paper and Supplementary Fig. 5 in the supplement.

SUPPLEMENTARY NOTE 7: PARITY SWITCHING POWER SPECTRA FOR ALL QUBITS

We implement a Ramsey pulse sequence that has been used previously to map QP parity onto qubit 1-state occupation [8–10]). We apply an $X/2$ pulse, idle for a time corresponding to a quarter of a qubit precession period, then apply a $Y/2$ pulse, followed by a qubit measurement. If the offset charge corresponds to the point of maximum charge dispersion, the final $Y/2$ pulse will rotate the state vector to the north/south poles of the Bloch sphere dependent on the QP parity state. Although some of the qubits on each chip have connections to the charge-bias line,

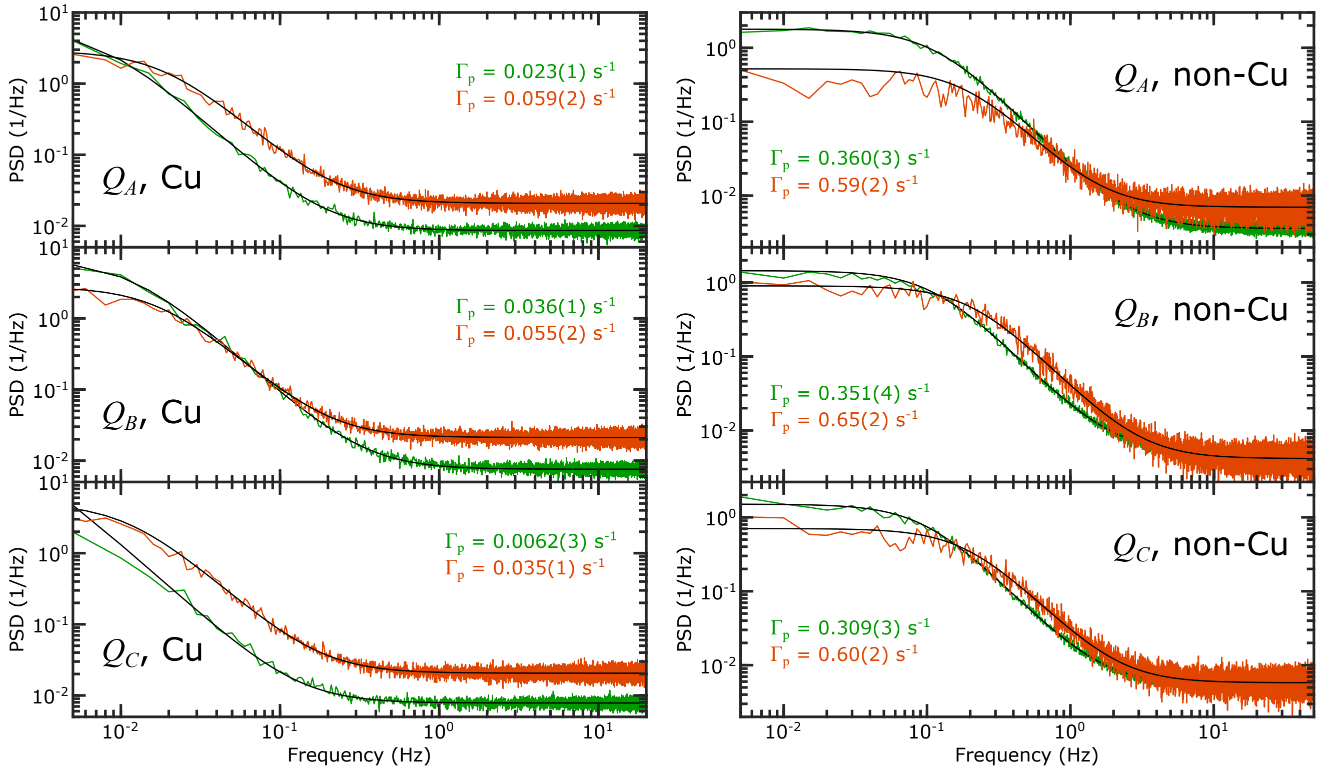

Supplementary Figure 7. **QP parity switching power spectra.** Plots of power spectral densities of QP parity switching for all qubits measured on the first (green) and second (orange) cooldowns; black lines correspond to PSD fits using Supplementary Eq. (1). The error bars on Γ_p values represent 95% confidence intervals from the fits.

we have chosen to perform our QP parity switching measurements without active stabilization of the offset charge. This allows the QP parity measurements to proceed without interruptions from periodic charge-tomography sequences [10]. However, when the offset charge jumps to near $(n + 1/2)e$ (for integer n), where the bands cross, the fidelity of the QP parity-mapping sequence approaches zero.

In order to compute the power spectral density of the QP parity switching, we perform the QP parity switching measurement on each qubit with 20,000 single shots at a repetition period of 10 ms (although PSD measurements for the Cu chip on the second cooldown used a 25-ms repetition period). For each single-shot measurement stream, we apply a simple thresholding based on the 0/1 readout calibration levels for each respective qubit to produce a digital time trace of QP parity. We then compute the PSD from the resulting digital trace and average several such PSD traces together (between 20-160) to obtain the curves in Fig. 3(a) in the main paper and Supplementary Fig. 7. Because we are not actively stabilizing the offset charge at the point of maximum dispersion, some of the PSD traces that are being averaged will have the environmental offset charge near the degeneracy point, where the QP parity readout fidelity vanishes. This results in an enhancement of the white noise floor, but still allows for a clear extraction of the characteristic QP parity switching rate.

We are able to fit the resulting power spectra with a single Lorentzian using the form described in Ref. [8]:

$$S_p(f) = \frac{4F^2\Gamma_{\mathrm{p}}}{(2\Gamma_{\mathrm{p}})^2 + (2\pi f)^2} + (1 - F^2)\Delta t, \tag{1}$$

where Γ_{p} is the parity switching rate, F is the parity sequence mapping fidelity, and Δt is the parity measurement repetition period.

Supplementary Fig. 7 shows the PSD for all three qubits on each chip. During our experiment, after collecting a majority of our data once the dilution refrigerator had been cold for several months, an unplanned power outage caused our dilution refrigerator to warm up to room temperature. Upon immediately cooling the same two devices back down, without making any changes to the wiring or shielding, we remeasured the PSD for each qubit within a few weeks of the start of this second cooldown. The plots in Supplementary Fig. 7 contain the PSD for each qubit on both chips measured on the first and second cooldowns. For all qubits, the QP parity switching rates increase on the second cooldown, likely because some elements in the qubit environment, for example, the isolators, attenuators, or shields, have not yet fully cooled to the base temperature (see Supplementary Note 12 for further discussion). Nonetheless, the Γ_{p} values on the Cu chip remain at least one order of magnitude lower compared to the non-Cu chip.

SUPPLEMENTARY NOTE 8: QP PARITY SWITCHING WITH PULSED INJECTION FOR ALL QUBITS

To complement Fig. 3(b) in the main paper, in this section, we plot the measured switching probability for each qubit on the non-Cu chip [Supplementary Fig. 8(a)] and Cu chip [Supplementary Fig. 8(b)] as a function of the injector junction pulse length. For each chip, all three qubits exhibit a similar behavior. Supplementary Fig. 8(c,d) contain the double- and triple-coincidence switching probabilities for the non-Cu and Cu chips, along with comparisons to the square and cube of the single-qubit switching probabilities for one of the qubits on each chip, as discussed in the main paper.

SUPPLEMENTARY NOTE 9: IDENTIFICATION OF QP PARITY SWITCHING EVENTS

In order to locate the parity switching steps from the simultaneous QP parity measurements, we apply the following data processing steps. First, because the offset charge was not actively stabilized, we need to identify the portions of the data stream for each qubit where the environmental offset charge jumped to near the degeneracy point, where the parity mapping fidelity approaches zero. This involves finding the envelope of the peak-to-peak signal for the parity time trace with an applied moving average of 100 time steps. If the envelope is below a threshold determined by the qubit 0/1 readout calibration levels, the portion of data until the envelope extends above the threshold is masked off and not analyzed further when digitizing the parity time traces. We next digitize the parity time traces by applying a moving average to the unmasked raw parity data to improve the signal-to-noise ratio. We then use a hidden Markov model (HMM) to identify the parity states. For the QP parity data without junction injection presented here, we use a moving average of 40 time steps. After fitting Gaussians to the qubit 0/1 single-shot readout calibration measurements, we use these distributions to assign a probability for the parity signal to have a value along the signal axis corresponding to an odd- or even-parity state. For the HMM, we also set the probability for the system to

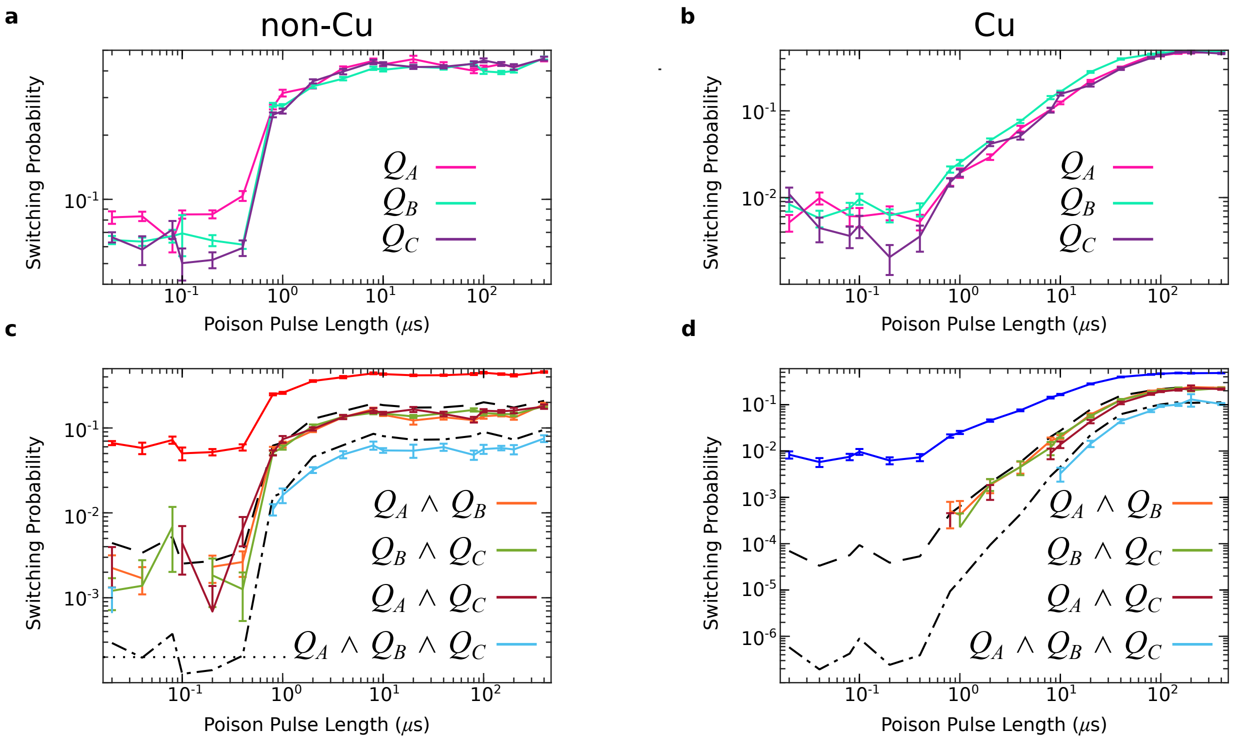

Supplementary Figure 8. **QP parity measurements with controlled injection for all qubits.** The switching probability, given by the measured parity switching rate divided by the pulse rate of the injector junction (20 Hz) vs. poison pulse duration for all three qubits on the (a) non-Cu and (b) Cu chips. Injection pulse amplitude V_b is 1 mV for both sets of measurements. The probability of double and triple coincidence events for the (c) non-Cu and (d) Cu samples are shown in comparison to the single switching probabilities [Q_C on non-Cu chip (red), Q_B on Cu chip (blue)] and the square (dash) and cube (dot-dash) of the single switching probabilities. The expected random background probability for two-fold coincidences on the non-Cu chip is indicated by the dotted line in (c). Error bars computed from standard Poisson counting errors.

transition from odd to even parity and vice versa based on the repetition time of the single shots and the Γ_p extracted separately from the QP parity PSDs for each qubit. With this information, we then use the Viterbi algorithm to fit a digital signal to the averaged data, thus extracting the most probable parity state given the readout value along the signal axis. In a few instances, we use a modified HMM scheme for the parity analysis. This involves implementing a simple threshold method which assigns the parity of the state based on the data with an applied moving average relative to the total mean of the data. With a parity value assigned at each time index, we derive the statistics for the value of the parity signal given its state. We then use these parameters and the transition probabilities described previously to augment the HMM approach and fit a digital signal to the averaged data. Supplementary Fig. 9 shows an example of ths parity switching analysis for Q_A on the non-Cu chip.

We then use the digital signal that was found through the HMM scheme to locate parity switches. We take the absolute value of the difference of adjacent points of the digital signal, which results in a peak at the location of each parity switch. The parity switching rate for each qubit r_i, where $i = A, B, C$, is given by N_i/τ_i, where N_i is the total number of parity switches for that particular qubit and τ_i is the total duration of unmasked data for the qubit. The uncertainty in r_i comes from the standard Poisson counting errors $N_i^{1/2}/\tau_i$.

SUPPLEMENTARY NOTE 10: EXTRACTION OF QP PARITY SWITCHING COINCIDENCES

Measuring the parity of all three qubits on either chip simultaneously allows us to track correlated events between qubits. In order to identify parity switching coincidences, we must process the digital parity traces obtained as described in the previous section to look for simultaneous switching between qubits. Because the moving averages that are applied to the raw parity measurement data to improve the signal-to-noise ratio also cause the switching events to have a shallower step, we must implement a windowing process to find coincidences. Because the effective

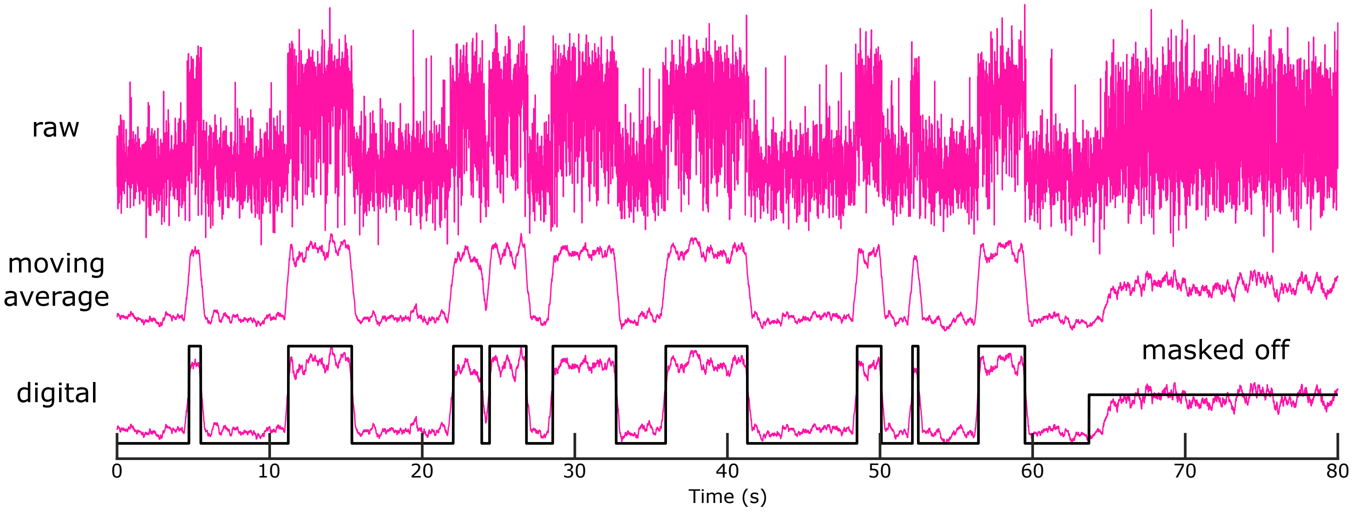

Supplementary Figure 9. **Identification of QP parity switching events.** Starting with the raw data at the top, a moving average over 40 time steps is then applied (middle); this is then converted to a digital signal using an HMM approach, shown as the black line on top of the averaged data in the trace at the bottom. For the final ~17 s, the environmental offset charge for the qubit was near charge degeneracy. The analysis code accounts for this by masking off this portion of the data, which is reflected in the digital signal displayed halfway between the levels for the different parities. This data is taken from Q_A for the non-Cu chip.

width of the switching steps is approximately equal to the number of moving average time steps, we set our window size to match the number of moving averages, thus, coincident switches should occur no farther apart than the width of the falling/rising edges.

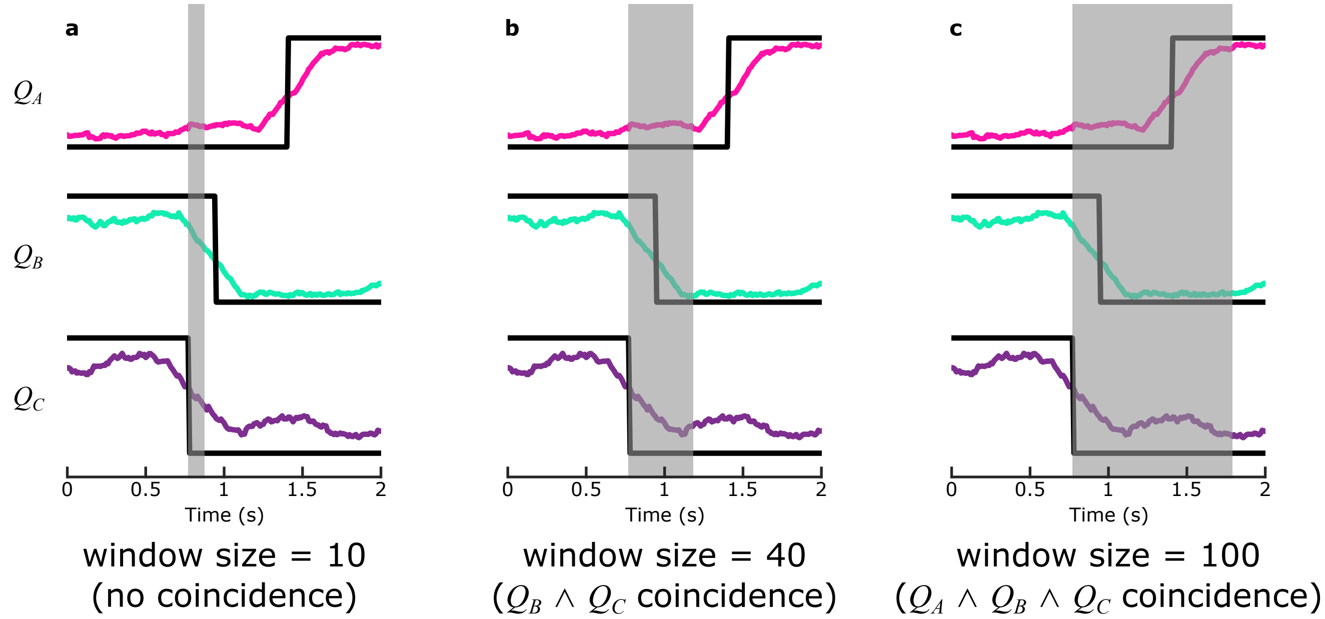

Supplementary Figure 10. **Data windowing for coincidence identification.** Example section of simultaneous QP parity data for non-Cu chip for a moving average over 40 time steps, with different window sizes applied. Window size indicated by shaded gray rectangle of (a) 10 time steps, resulting in no identified coincidences; (b) 40 time steps, correctly identifying a $Q_B \wedge Q_C$ coincidence; (c) 100 time steps, misidentifying a $Q_A \wedge Q_B \wedge Q_C$ coincidence.

Supplementary Fig. 10 shows the effects of different window sizes for the same example parity data trace. We sweep our window through the simultaneous digital signals, and if multiple switches occur within our window size, they are identified as coincidences. For a window size well below the number of moving averages, the code misses a double

coincidence [Supplementary Fig. 10(a)], while for a window size much greater than the number of moving averages, switches from separate events are misidentified as a coincidence.

Following the coincidence switching identification, the events are indexed with the appropriate type ($Q_A \wedge Q_B$, $Q_B \wedge Q_C$, $Q_A \wedge Q_C$, or $Q_A \wedge Q_B \wedge Q_C$). With this approach, every triple coincidence is also counted as three double coincidences. We also restrict each switch of a given qubit to participate in only one event per coincidence type. For example, a Q_B switch cannot be used for two $Q_A \wedge Q_B$ coincidences, but could be used for a $Q_A \wedge Q_B$ coincidence and a $Q_B \wedge Q_C$ coincidence. In Supplementary Fig. 11, we present example simultaneous parity traces for all three qubits for both chips. We also represent the locations of extracted coincidences with vertical dashed lines.

The switching rate for each type of coincidence event r_i, where $i = AB, BC, AC, ABC$, is given by N_i/τ_i, where N_i is the total number of events and τ_i is the total duration of unmasked data for event type i. Note that double coincidences between qubits j and k are only counted during the period when both qubits are unmasked; similarly, triple coincidences require that all three qubits are unmasked. The uncertainty in r_i comes from the standard Poisson counting errors $N_i^{1/2}/\tau_i$.

In Supplementary Table 2, we explore the effect of different window sizes and moving averages on the observed parity switching rates for both chips. For higher averages, we observe a moderate decrease (∼10%) in the single-qubit switching rate, which we attribute to occasional narrow features with two closely spaced switches that get averaged below the threshold for larger numbers of moving averages. At the same time, the double- and triple-coincidence rates increase somewhat as the window size increases. Nonetheless, we still observe the same overall trend between the two chips: the single-qubit parity switching rates for the Cu chip remain ∼1 order of magnitude lower than for the non-Cu chip, and the double- and triple-coincidence rates are still ∼2 orders of magnitude lower.

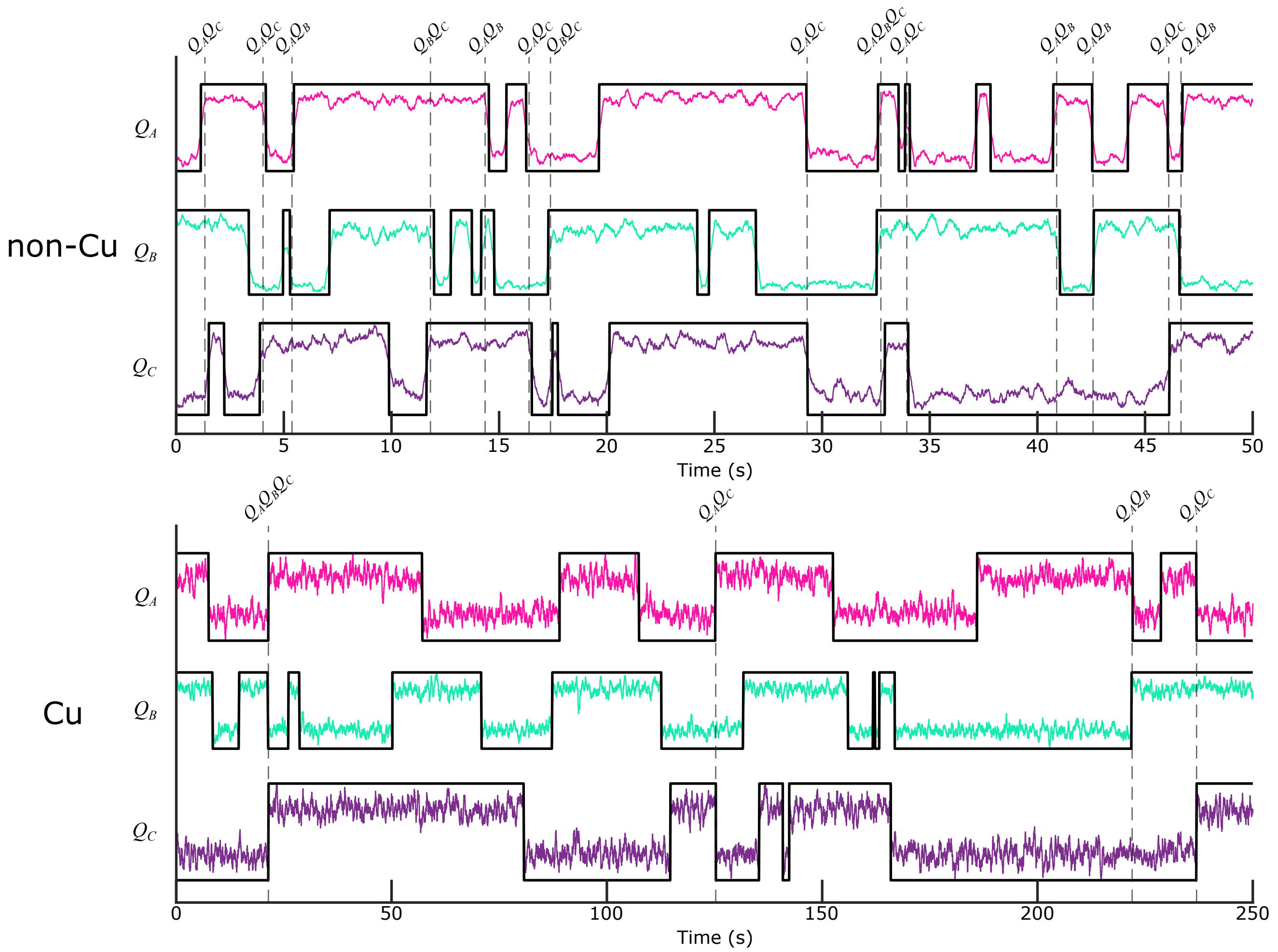

Supplementary Figure 11. **Identification of QP parity switching coincidences for both chips.** Examples of extracted digital QP parity signals from simultaneous QP parity data and identification of coincidences. Note the 5x difference in the timespans between the bottom plot for the Cu chip and the upper plot for the non-Cu chip.

Observed, random background, and extracted parity switching rates ($s^{-1} \times 10^{-3}$) for different window sizes and moving averages										
Device	window size, moving average	Type	Q_A	Q_B	Q_C	$Q_A \wedge Q_B$	$Q_B \wedge Q_C$	$Q_A \wedge Q_C$	$Q_A \wedge Q_B \wedge Q_C$
non-Cu	20, 20	observed	320(3)	333(4)	272(3)	42(2)	38(2)	36(1)	6.1(8)
		background	-	-	-	21.3(4)	18.1(3)	17.4(3)	1.16(2)
		extracted	410(20)	430(20)	320(20)	110(10)	100(10)	100(10)	23(8)
	20, 40	observed	299(3)	301(4)	252(3)	37(2)	35(2)	33(1)	5.2(8)
		background	-	-	-	18.0(3)	15.2(3)	15.0(2)	0.91(2)
		extracted	390(20)	390(20)	300(20)	100(10)	100(10)	90(10)	20(7)
	40, 40	observed	299(3)	301(4)	252(3)	65(2)	60(2)	57(2)	12(1)
		background	-	-	-	36.0(6)	30.3(5)	30.1(5)	3.62(8)
		extracted	200(20)	190(20)	120(20)	180(10)	170(10)	150(10)	64(9)
Cu	20, 20	observed	25.9(4)	36.5(5)	32.7(7)	0.56(8)	0.6(2)	0.5(1)	0.06(6)
		background	-	-	-	0.189(4)	0.239(6)	0.169(4)	0.00124(4)
		extracted	49(1)	70(1)	63(2)	1.1(7)	1.2(9)	0.7(7)	0.5(5)
	20, 40	observed	22.1(3)	33.6(5)	23.0(5)	0.57(8)	0.4(1)	0.36(9)	0.06(6)
		background	-	-	-	0.149(3)	0.155(4)	0.102(3)	0.00069(2)
		extracted	42(1)	65(1)	44(1)	1.2(7)	0.7(8)	0.6(7)	0.5(5)
	40, 40	observed	22.1(3)	33.6(5)	23.0(5)	0.8(1)	0.8(2)	0.5(1)	0.06(6)
		background	-	-	-	0.298(6)	0.310(9)	0.204(6)	0.00274(9)
		extracted	41(1)	63(1)	43(2)	1.9(7)	1.6(9)	1.0(7)	0.4(6)

Supplementary Table 2. **Comparison of rates for different windowing and averaging.** Observed switching rates, random background coincidence rates, and extracted poisoning event rates for different window size and moving average combinations across both chips. The entries for window size = 40 and moving average = 40 match those in Fig. 4(b) of the main paper. Note the scale factor of 10^{-3} on the rate units.

SUPPLEMENTARY NOTE 11: IDENTIFICATION OF CORRELATED QP POISONING RATES

For a set of observed single-qubit parity switching rates $r_{A-C}^{\rm obs}$ with a non-zero window Δt for identifying double- and triple-coincidence switching, one would expect a rate for random uncorrelated coincidence switching given by the product of the probabilities for observing a parity switch for each of the constituent qubits in the coincidence event during the interval Δt. Thus, the expected random background double-concidence rate for qubits i and j is given by $(r_i^{\rm obs}\Delta t)(r_j^{\rm obs}\Delta t)/\Delta t$; similarly the expected random background triple-coincidence rate for qubits i, j, and k is given by $(r_i^{\rm obs}\Delta t)(r_j^{\rm obs}\Delta t)(r_k^{\rm obs}\Delta t)/\Delta t$. These expected random coincidence parity switching rates are listed in Supplementary Table 2 for different numbers of moving averages and window sizes. The error bars for these random background coincidence rates were computed by summing the fractional uncertainty for each observed rate in quadrature. We note that these random background coincidence rates remain well below the observed rates as we vary the windowing and averaging.

While the quantities we measure in our simultaneous parity measurements are the observed parity switching rates, we would like to compute the actual poisoning event rates for each qubit, or group of qubits in the case of correlated poisoning. This calculation requires accounting for the random background coincidence switching described above, as well as the probability for recording a parity switch for a given poisoning event: 1/2 in the case of single-qubit poisoning, 1/4 for double-qubit correlated poisoning, and 1/8 for triple-qubit poisoning, as described in the main paper.

For the observed parity switching, each double-coincidence event will also be recorded as two single-qubit switching events; similarly, each triple-coincidence event will also be recorded as three double-qubit switching events and three single-qubit switching events. Here, we define the extracted poisoning event rates r_i to be exclusive; for example, a single poisoning event that couples to both Q_A and Q_B will contribute to r_{AB} but will not contribute to r_A or r_B.

Based on these criteria, we can use the observed parity switching rates $r_i^{\rm obs}$ to compute the probability for observing each type of parity switching event in a window interval Δt as $p_i^{\rm obs} = r_i^{\rm obs}\Delta t$. We can then derive expressions for the probability of observing each type of parity switching event in terms of the actual probability for each type of

Observed parity switching and extracted poisoning event rates							
Device	Qubit(s)	Separation	N_i	τ_i (s)	r_i^{obs} (s^{-1})	$r_i^{\mathrm{background}}$ (s^{-1})	r_i (s^{-1})
non-Cu	Q_A	-	8528	28,557	0.299(3)	-	0.20(2)
	Q_B	-	5202	17,272	0.301(4)	-	0.19(2)
	Q_C	-	7959	31,609	0.252(3)	-	0.12(2)
	$Q_A \wedge Q_B$	5.3 mm	832	12,851	0.065(2)	0.0360(6)	0.18(1)
	$Q_B \wedge Q_C$	4.5 mm	670	11,124	0.060(2)	0.0303(5)	0.17(1)
	$Q_A \wedge Q_C$	2.0 mm	1078	18,941	0.057(2)	0.0301(5)	0.15(1)
	$Q_A \wedge Q_B \wedge Q_C$	-	109	8,842	0.012(1)	0.00362(8)	0.064(9)
Cu	Q_A	-	4031	182,103	0.0221(3)	-	0.041(1)
	Q_B	-	4515	134,192	0.0336(5)	-	0.063(1)
	Q_C	-	1779	77,322	0.0230(5)	-	0.043(2)
	$Q_A \wedge Q_B$	5.3 mm	66	78,936	0.0008(1)	0.000298(6)	0.0019(7)
	$Q_B \wedge Q_C$	4.5 mm	20	25,376	0.0008(2)	0.000310(9)	0.0016(9)
	$Q_A \wedge Q_C$	2.0 mm	22	41,277	0.0005(1)	0.000204(6)	0.0010(7)
	$Q_A \wedge Q_B \wedge Q_C$	-	1	15,389	0.00006(6)	0.00000274(9)	0.0004(6)

Supplementary Table 3. **Summary of observed, background, and extracted rates.** Observed number of switches and total measurement time leading to observed switching rates r_i^{obs}, expected random background coincidence rates $r_i^{\mathrm{background}}$, and extracted poisoning event rates r_i for each qubit and qubit combination across both chips for 40 moving averages and a window size of 40, corresponding to the rates plotted in Fig. 4(b) of the main paper.

poisoning event:

$$\begin{aligned}
p_A^{\mathrm{obs}} &= \frac{1}{2}\left(p_{ABC} + p_{AB} + p_{AC} + p_A\right) \\
p_B^{\mathrm{obs}} &= \frac{1}{2}\left(p_{ABC} + p_{AB} + p_{BC} + p_B\right) \\
p_C^{\mathrm{obs}} &= \frac{1}{2}\left(p_{ABC} + p_{AC} + p_{BC} + p_C\right) \\
p_{AB}^{\mathrm{obs}} &= \frac{1}{4}\left(p_{ABC} + p_{AB} + p_A p_B\right) \\
p_{BC}^{\mathrm{obs}} &= \frac{1}{4}\left(p_{ABC} + p_{BC} + p_B p_C\right) \\
p_{AC}^{\mathrm{obs}} &= \frac{1}{4}\left(p_{ABC} + p_{AC} + p_A p_C\right) \\
p_{ABC}^{\mathrm{obs}} &= \frac{1}{8}\left(p_{ABC} + p_A p_B p_C + p_{AB} p_C + p_A p_{BC} + p_{AC} p_B\right).
\end{aligned} \tag{2}$$

With the experimentally measured switching probabilities p_i^{obs}, we numerically solve the system of equations to obtain the actual poisoning probabilities p_i. We then calculate the actual poisoning rates $r_i = p_i/\Delta t$. We compute the error bars on each actual poisoning probability by numerically computing the derivative with respect to each of the observed switching probabilities, then multiplying by the corresponding Poisson error bar for the observed switching probability and summing these together in quadrature.

In Supplementary Table 3, we list the values N_i for each single qubit parity switch and coincidence event, as well as the total unmasked duration τ_i for the particular type of event. For the right three columns, the observed parity switching rates r_i^{obs}, expected random background coincidence rates $r_i^{\mathrm{background}}$, and extracted actual poisoning rates r_i correspond to the values presented in Fig. 4(b) in the main paper.

SUPPLEMENTARY NOTE 12: QP PARITY SWITCHING RATES ON DIFFERENT COOLDOWNS

As described in Supplementary Note 7, an unplanned power outage caused our experiment to be split between two cooldowns. Most of the data was collected during the first cooldown, after the dilution refrigerator had been cold for several months. Data measured during the second cooldown was taken within a few weeks of the start of the cooldown. Supplementary Table 4 compares the observed parity switching rates and extracted poisoning rates for the non-Cu chip on the two cooldowns. Supplementary Table 5 makes the same comparison for the Cu chip. Although the cryostat was not opened in between the cooldowns and nothing was changed in the wiring, filtering, or shielding,

the shorter time period after the start of the second cooldown likely resulted in incomplete thermalization of the radiative environment of the qubit, potentially involving amorphous, non-metallic elements in some of the microwave components or qubit packaging that could slowly release heat over long timescales. This would lead to higher effective blackbody temperatures and a larger flux of THz photons or enhancements to other sources of pair-breaking phonons, such as heat-only events [11–13], thus resulting in the higher poisoning rates observed on both chips. We note that Ref. [14] also reported a slow decay in QP poisoning rates of a mesoscopic superconducting island over a timescale of several weeks with no clear mechanism for the source of the poisoning.

Observed parity switching rates (s^{-1}) for the non-Cu chip							
**Cooldown**	Q_A	Q_B	Q_C	$Q_A \wedge Q_B$	$Q_B \wedge Q_C$	$Q_A \wedge Q_C$	$Q_A \wedge Q_B \wedge Q_C$
1	0.299(3)	0.301(4)	0.252(3)	0.065(2)	0.060(2)	0.057(2)	0.012(1)
2	0.505(5)	0.508(4)	0.495(8)	0.170(4)	0.161(6)	0.162(8)	0.042(5)
Extracted poisoning event rates (s^{-1})							
1	0.20(2)	0.19(2)	0.12(2)	0.18(1)	0.17(1)	0.15(1)	0.064(9)
2	0.01(4)	0.02(4)	0.03(5)	0.35(3)	0.31(3)	0.32(4)	0.33(3)
spacing	-	-	-	5.3 mm	4.5 mm	2.0 mm	-

Supplementary Table 4. **Comparison of rates for non-Cu chip between cooldowns.** Observed parity switching rates and extracted poisoning event rates for non-Cu chip on the first and second cooldowns with no poisoning from injector junction.

Observed parity switching rates (s^{-1}) for the Cu chip							
Cooldown	Q_A	Q_B	Q_C	$Q_A \wedge Q_B$	$Q_B \wedge Q_C$	$Q_A \wedge Q_C$	$Q_A \wedge Q_B \wedge Q_C$
1	0.0221(3)	0.0336(5)	0.0230(5)	0.0008(1)	0.0008(2)	0.0005(1)	0.00006(6)
2	0.056(2)	0.053(2)	0.039(1)	0.0051(9)	0.005(1)	0.0047(7)	0.0003(3)
Extracted poisoning event rates (s^{-1})							
1	0.041(1)	0.063(1)	0.043(2)	0.0019(7)	0.0016(9)	0.0010(7)	0.0004(6)
2	0.082(8)	0.07(1)	0.047(8)	0.015(6)	0.017(6)	0.016(5)	0.000(3)*
spacing	-	-	-	5.3 mm	4.5 mm	2.0 mm	-

Supplementary Table 5. **Comparison of rates for Cu chip between cooldowns.** Observed parity switching rates and extracted poisoning event rates for Cu chip on the first and second cooldowns with no poisoning from injector junction. *For the Cu chip on the second cooldown, the solution to the system of equations in Supplementary Eq. (2) results in a small negative value for the three-fold coincidence poisoning event rate that is consistent with zero based on the calculated uncertainty.

SUPPLEMENTARY NOTE 13: OFFSET CHARGE MEASUREMENTS

For a charge-sensitive qubit, besides the parity-mapping sequence, one can also perform a charge tomography sequence to measure the environmental offset charge, provided the qubit has a charge-bias line [10, 15]. The Ramsey sequence involves two $X/2$ pulses with an idle time $t_i = 1/2\delta f$, where $2\delta f$ is the maximum charge dispersion for the qubit. A qubit measurement at the end of the sequence results in a 1-state probability:

$$P_1 = \frac{1}{2}\left[d + \nu \cos\left(\pi \cos 2\pi n_g\right)\right], \tag{3}$$

where n_g is the sum of the externally applied gate charge n_g^{ext} and the environmental offset charge δn_g; d and ν are fitting parameters. Supplementary Fig. 12(a) shows an example charge tomography trace for Q_B on the non-Cu chip and a fit to Supplementary Eq. (3).

The charge tomography measurement sequence takes 28 (28.8) s and we repeat this sequence 2000 (2250) times for Q_B (Q_A) on the non-Cu (Cu) chip. (Q_B on the non-Cu chip; Q_A on the Cu chip). From the fit to each tomography scan, we extract δn_g, which we plot as a function of time over 16 (18) hours for the non-Cu (Cu) chips [Supplementary Fig. 12(b)]. From these traces, we find that large charge jumps ($\Delta q > 0.1e$) occur at a rate of 0.0012(1) s^{-1} and 0.0011(1) s^{-1} for a qubit on the Cu and non-Cu chips, respectively.

Based on this rate of offset charge jumps, we can estimate the rate of γ impacts on the chip R_γ by following the detailed analysis in Ref. [15]. In this case, the authors obtained $R_\gamma = 0.0198$ s^{-1} from similar measurements of offset charge jump rates, combined with detailed modeling of the effective charge sensing area of their qubits (19,902 μm^2) and simulations of the charge dynamics in the Si substrate. We can approximate the charge sensing area for our

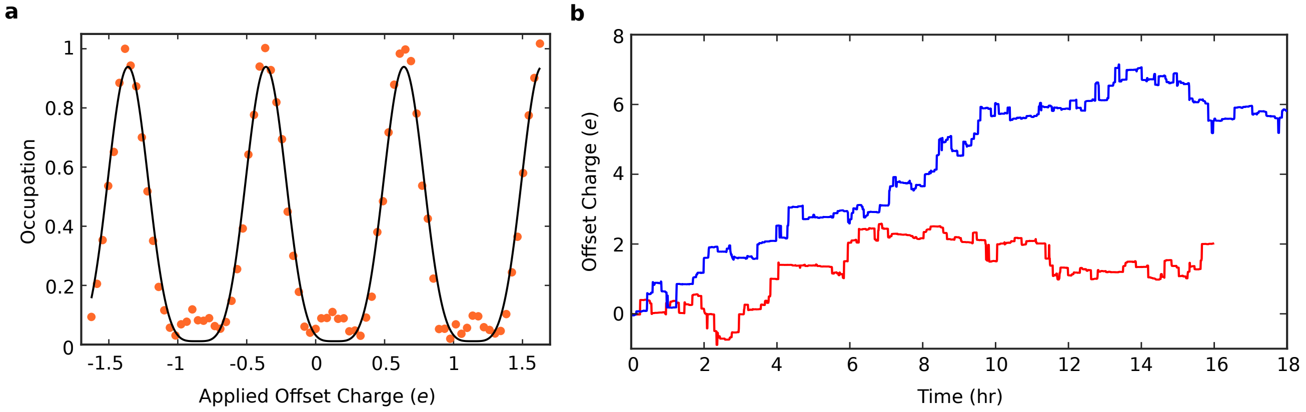

Supplementary Figure 12. **Offset charge measurements.** (a) Example charge tomography measurement (orange) and fit to Supplementary Eq. (3) (black) for Q_B on the non-Cu chip. (b) Offset charge vs. time for Q_B on the non-Cu chip (red) and Q_A on the Cu chip (blue).

qubits by taking this to be the area of the qubit shunt capacitor island extended out to half of the distance between the island and ground plane pocket (6612 μm^2). To estimate R_γ for our experiment, we scale the corresponding value in Ref. [15] by the ratio of the charge sensing area in Ref. [15] to that for our qubit, the ratio of our measured offset charge jump rate to that in Ref. [15] (0.00135 s^{-1}), and the ratio of our qubit chip area [(8 mm)^2] to that in Ref. [15] [(6.25 mm)^2], leading to the estimate $R_\gamma = 0.083(8)$ s^{-1} in our system.

SUPPLEMENTARY NOTE 14: EDITING OF DEVICE IMAGES

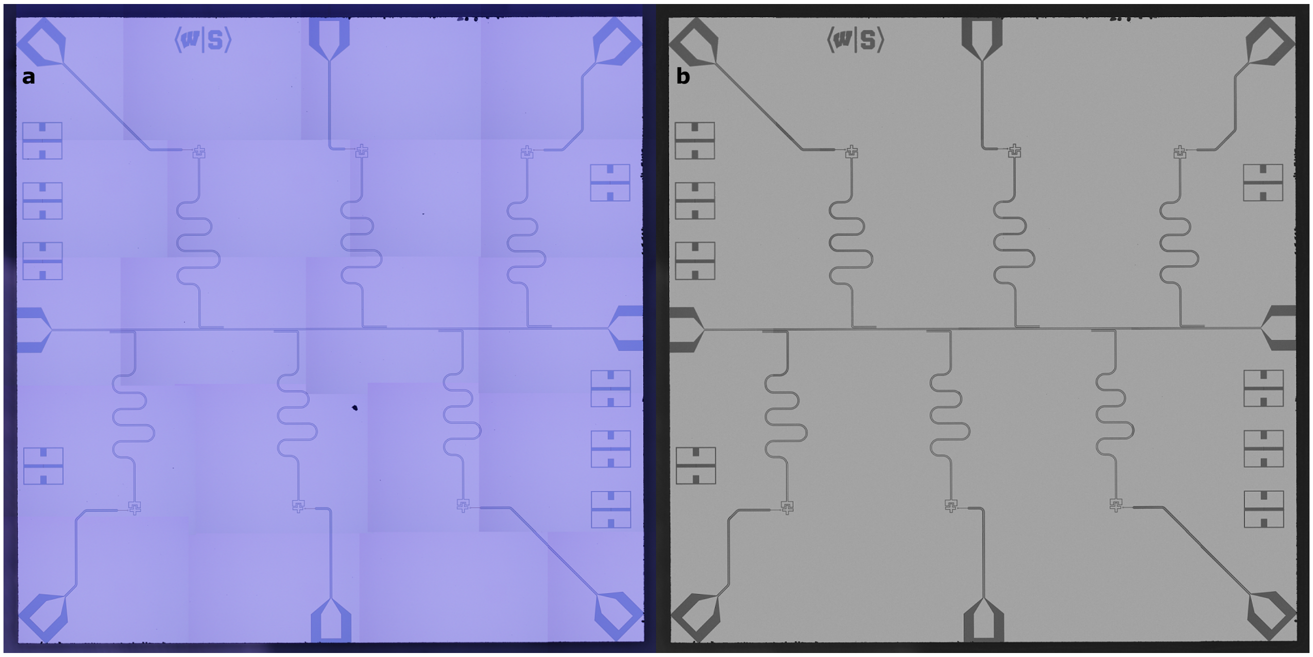

Supplementary Figure 13. **Raw device image editing.** (a) Tiled layout of the 20 raw images stitched together to make the full chip image. (b) Final chip image after editing.

The image presented in Supplementary Fig. 13(a) was made by stitching together 20 optical micrographs to achieve a full chip picture. Once each micrograph was aligned, the composite image was converted to grayscale and the contrast increased. At this step, minor surface contamination was removed digitally to limit distraction from important device

features. Finally, to remove the vignetting present in each individual picture, the image was processed using MATLAB, which identified the range of pixel values for the Nb background and altered each pixel to reflect the average value with some random noise. The result can be seen in Supplementary Fig. 13(b). The images presented in Fig. 1 and Supplementary Fig. 1 have been false-colored to highlight different parts of the chip.

* These authors contributed equally
† bplourde@syr.edu

[1] Barends, R. *et al.* Minimizing quasiparticle generation from stray infrared light in superconducting quantum circuits. *Applied Physics Letters* **99**, 113507 (2011).
[2] Wang, C. *et al.* Measurement and control of quasiparticle dynamics in a superconducting qubit. *Nature Communications* **5**, 1–7 (2014).
[3] Vernon Jr, F. L. & Pedersen, R. J. Relaxation oscillations in Josephson junctions. *Journal of Applied Physics* **39**, 2661–2664 (1968).
[4] Rafferty, O. *et al.* Spurious antenna modes of the transmon qubit. *arXiv preprint arXiv:2103.06803* (2021).
[5] Liu, C.-H. *et al.* Quasiparticle poisoning of superconducting qubits from resonant absorption of pair-breaking photons. *arXiv preprint arXiv:2203.06577* (2022).
[6] Houzet, M., Serniak, K., Catelani, G., Devoret, M. & Glazman, L. Photon-assisted charge-parity jumps in a superconducting qubit. *Physical Review Letters* **123**, 107704 (2019).
[7] CST Studio Suite. www.3ds.com.
[8] Ristè, D. *et al.* Millisecond charge-parity fluctuations and induced decoherence in a superconducting transmon qubit. *Nature Communications* **4**, 1–6 (2013).
[9] Serniak, K. *et al.* Hot nonequilibrium quasiparticles in transmon qubits. *Physical Review Letters* **121**, 157701 (2018).
[10] Christensen, B. *et al.* Anomalous charge noise in superconducting qubits. *Physical Review B* **100**, 140503 (2019).
[11] Åström, J. *et al.* Fracture processes observed with a cryogenic detector. *Physics Letters A* **356**, 262–266 (2006).
[12] Armengaud, E. *et al.* Constraints on low-mass WIMPs from the EDELWEISS-III dark matter search. *Journal of Cosmology and Astroparticle Physics* **2016**, 019 (2016).
[13] Anthony-Petersen, R. *et al.* A stress induced source of phonon bursts and quasiparticle poisoning. *arXiv preprint arXiv:2208.02790* (2022).
[14] Mannila, E. T. *et al.* A superconductor free of quasiparticles for seconds. *Nature Physics* **18**, 145–148 (2022).
[15] Wilen, C. *et al.* Correlated charge noise and relaxation errors in superconducting qubits. *Nature* **594**, 369–373 (2021).
